# 26*S*蛋白酶体亚基在非小细胞肺癌中的研究及治疗进展

**DOI:** 10.3779/j.issn.1009-3419.2025.106.13

**Published:** 2025-05-20

**Authors:** Chenrui MOU, Shaotong ZOU, Chao REN, Zihan YI, Jianlin SHI

**Affiliations:** ^1^650118 昆明，昆明医科大学第三附属医院（云南省肿瘤医院）胸外科; ^1^Department of Chest Surgery, The Third Affiliated Hospital of Kunming Medical University, Yunnan Tumor Hospital, Kunming 650118, China; ^2^650032 昆明，昆明医科大学; ^2^Kunming Medical University, Kunming 650032, China

**Keywords:** 肺肿瘤, 26*S*蛋白酶体, 19*S*调节颗粒, 20*S*核心颗粒, Lung neoplasms, 26*S* proteasome subunits, 19*S* regulatory particle, 20*S* core particle

## Abstract

肺癌是全球最常见的癌症之一，也是癌症死亡的第一大原因。肺腺癌是最常见的肺癌类型。由于在肺腺癌的增殖和转移过程中缺乏有效的生物标志物及治疗靶点，肺腺癌的总体治疗不容乐观。因此，需要寻找肺腺癌治疗的新思路及方法。26*S*蛋白酶体是一种负责降解错误折叠蛋白并维持细胞内蛋白质稳态的多蛋白复合物。在非小细胞肺癌（non-small cell lung cancer, NSCLC）的发展过程中，26*S*蛋白酶体的调节颗粒亚基通过调节肿瘤相关蛋白、免疫细胞以及相关信号通路，促进肿瘤的恶性进展。蛋白酶体核心颗粒是降解蛋白质的关键亚基，其抑制剂在与常规化疗药物联合使用时显示出良好的抗肿瘤效果。然而，受限于毒副作用和肿瘤异质性，目前针对26*S*蛋白酶体的靶向抑制剂在NSCLC治疗中的应用仍不广泛。本文综述了26*S*蛋白酶体调节颗粒亚基和核心颗粒亚基在NSCLC的作用机制及相关治疗研究，探讨了这些抑制剂在临床应用中的潜力。

## Abstract (en)

Lung cancer is one of the most common cancers worldwide and is the leading cause of cancer deaths. Lung adenocarcinoma is the most common type of lung cancer. Due to the lack of effective biomarkers and therapeutic targets in the proliferation and metastasis of lung adenocarcinoma, the overall treatment of lung adenocarcinoma is not optimistic. Therefore, there is a need to find new ideas and methods for lung adenocarcinoma treatment. The 26*S* proteasome is a multiprotein complex responsible for degrading misfolded proteins and maintaining intracellular protein homeostasis. During the development of non-small cell lung cancer (NSCLC), the regulatory granule subunit of the 26*S* proteasome promotes the malignant progression of tumours by regulating tumour-associated proteins, immune cells, and related signalling pathways. The proteasome core particle is a key subunit for degrading proteins, and its inhibitors have shown promising anti-tumour effects when combined with conventional chemotherapeutic agents. However, limited by toxic side effects and tumour heterogeneity, targeted inhibitors against the 26*S* proteasome are still not widely used in NSCLC treatment. This article reviews the mechanism of action and related therapeutic research of 26*S* proteasome regulatory particle subunits and core particle subunits in NSCLC, and explores the potential of these inhibitors in clinical application.

肺癌是全世界范围内影响人类健康的重要疾病，据统计2022年肺癌新发病例和死亡率均居全球肿瘤第一位^[1]^。中国肺癌的发病率和死亡率均超过全球平均水平，在所有肿瘤中位居第一位^[2]^。从病理学的角度肺癌被划分为非小细胞肺癌（non-small cell lung cancer, NSCLC）和小细胞肺癌^[3]^，其中NSCLC占80%左右^[4]^。近年来，NSCLC的治疗方式包括手术切除、化疗、放疗、分子靶向及免疫治疗^[5]^，但由于个体差异、肿瘤异质性、药物的毒副作用和耐药等问题，使得患者平均5年生存率仍不足20%^[6]^。因此，寻找新的肿瘤标志物和有效的药物治疗靶点对NSCLC患者的早期诊断、精准治疗及预后具有重要意义。

泛素蛋白酶体系统（ubiquitin-proteasome system, UPS）是真核细胞中降解错误折叠蛋白质的主要途径^[7]^。该系统由泛素、泛素酶、去泛素化酶以及26*S*蛋白酶体组成^[8]^。26*S*蛋白酶体凭借其独特的结构及功能，在维持蛋白质稳态和调控细胞过程等方面发挥着重要作用^[9]^。受基因组不稳定性和快速增殖性等因素影响，恶性肿瘤在发生、发展过程中可产生大量破坏及错误折叠的蛋白质，这对肿瘤细胞而言往往是致命的^[10]^。为缓解这一蛋白毒性压力，肿瘤细胞内26*S*蛋白酶体活性普遍增强^[11]^。因此，抑制26*S*蛋白酶体的活性可为NSCLC的治疗提供新的思路^[12]^。然而，要实现26*S*蛋白酶体抑制剂在NSCLC治疗上的广泛应用，仍需要克服肿瘤异质性、药物安全性及耐药性等挑战。本文将综述26*S*蛋白酶体在NSCLC进展中的机制与功能，并展望其在NSCLC发展中的研究方向及临床应用。

## 1 26*S*蛋白酶体的分子结构和生物学功能

26*S*蛋白酶体是一种相对分子量为2.5 MDa的蛋白水解复合物。它由两部分组成，分别是19*S*调节颗粒和20*S*核心颗粒^[13]^。20*S*核心颗粒由4个堆叠的七聚体环构成，形成α_1-7_-β_1-7_-β_1-7_-α_1-7_的立体结构。位于20*S*核心颗粒内部的蛋白酶体亚基β型5（proteasome subunit beta type 5, PSMB5）、PSMB6和PSMB7分别具有凝乳胰蛋白酶、半胱天冬酶及胰蛋白酶样蛋白水解活性^[14]^，是蛋白质降解的主要功能亚基。19*S*调节颗粒由“盖子”（lid）和“底座”（base）构成，并结合于20*S*核心颗粒的两端^[15]^。19*S*调节颗粒依赖腺苷三磷酸（adenosine triphosphate, ATP）水解供能，识别泛素标记的靶蛋白并将其转运至核心颗粒中，最终将蛋白质水解为小肽段^[16]^。目前越来越多的研究发现，26*S*蛋白酶体不仅是UPS降解异常蛋白质的关键环节^[17]^，还广泛参与多种生理过程，包括细胞周期、DNA复制、转录、信号传导、应激反应和炎症机制等^[13]^。研究^[18-21]^还表明，26*S*蛋白酶体通过影响肿瘤相关蛋白[如p53、p21和PTEN诱导激酶1（PTEN induced putative kinase, PINK1）等]及转录因子的降解过程，参与NSCLC侵袭转移、增殖和免疫逃逸等多种恶性生物学行为（表1）。

**表 1:** 与非小细胞肺癌发病机制相关的蛋白酶体亚基的名称及功能

Proteasome subunit	Another name	Change in NSCLC	Function	Reference
Rpn3	PSMD3	Upregulation	Inhibition of ILF3 degradation and regulation of proliferation and metastasis	^[22]^
Rpn5	PSMD12	Upregulation	Promotion of Nrf2 nuclear translocation; regulation of TrxR1 transcription and promotion of proliferation and metastasis	^[23]^
Rpn11	PSMD14	Upregulation	Degradation of p53 protein and inhibition of apoptosis	^[24]^
Rpn8	PSMD7	Upregulation	Degradation of p53 protein and promotion of proliferation	^[25]^
Rpn1	PSMD2	Upregulation	Regulation of p21 and immune cells to promote proliferation and metastasis	^[26,27]^
Rpn2	PSMD1	Upregulation	Regulation of ECM-receptors and immune cell infiltration	^[28]^
Rpt4	PSMC6	Upregulation	Promotion of proliferation by degrading Axin1 and activating the Wnt signalling pathway	^[29]^
PSMA7	-	Upregulation	Controversial	^[30]^

Rpn: regulatory particle non-ATPase subunit; PSMD: proteasome 26*S* subunit non-ATPase; ILF3: interleukin enhancer binding factor 3; Nrf2: nuclear factor E2 related factor; TrxR1: thioredoxin reductase 1; Rpt4: regulatory particle triple ATPase 4; PSMC6: proteasome 26*S* subunit ATPase 6; Axin1: axis inhibition protein 1; PSMA7: proteasome subunit *α*7.

## 2 19*S*调节颗粒亚基在NSCLC中的研究进展

“盖子”由9个调节颗粒非ATP酶亚基（regulatory particle non-ATPase subunit, Rpn）构成^[31]^，包括Rpn3、Rpn5-9、Rpn11、Rpn12和Rpn15^[32]^。除了Rpn15之外，其余亚基的C-末端具有较高的保守性，能够形成螺旋束，在盖子组装过程中发挥指导作用^[33]^。调节颗粒的“底座”由两种类型的亚基构成，一类由Rpn1、Rpn2、Rpn10和Rpn13组成，其中泛素受体Rpn1、Rpn10和Rpn13可以结合被泛素标记的蛋白；另一类是由6个不同的调节颗粒AAA ATP酶（regulatory particle triple ATPase, Rpt）构成的六聚杂环（Rpt1-Rpt2-Rpt6-Rpt3-Rpt4-Rpt5）。它们在ATP的帮助下，对泛素受体识别的底物施加“拉力”，并将其展开并转运到核心颗粒的肽酶腔中^[34]^。在此过程中，19*S*调节颗粒亚基（如Rpn1^[35]^、Rpn2^[36]^、Rpn3^[22]^、Rpn5^[23]^、Rpn8及Rpn11^[37]^）已被证实通过调控肿瘤相关蛋白稳定性及免疫微环境参与NSCLC恶性进展。

### 2.1 Rpn3与Rpn5促进NSCLC细胞增殖与转移

Rpn3位于染色体ch17q21区域，该区域与多种肿瘤的恶性进展相关^[38]^。研究^[22]^发现，NSCLC组织中Rpn3表达显著升高，且与肿瘤侵袭、转移及患者不良预后相关。白介素增强结合因子3（interleukin enhancer binding factor 3, ILF3）与表皮生长因子受体（epidermal growth factor receptor, EGFR）在NSCLC中高表达，并促进NSCLC的恶性进展，两者呈正相关关系^[39]^。Zhang等^[22]^研究发现，Rpn3通过抑制ILF3的降解，促进NSCLC细胞的增殖及转移。然而，Rpn3是否通过ILF3调控EGFR的表达，仍需要进一步研究。另有研究^[23]^表明，Rpn5通过抑制核因子E2相关因子2（nuclear factor E2 related factor, Nrf2）的降解并促进其核易位，激活硫氧还蛋白还原酶1（thioredoxin reductase 1, TrxR1）转录，从而促进NSCLC细胞的增殖和转移。

### 2.2 Rpn8与Rpn11通过促进p53蛋白的降解促进NSCLC细胞增殖

Rpn8与Rpn11是19*S*调节颗粒中具有去泛素化功能的关键亚基。它们通过形成异源二聚体，共同催化底物蛋白去泛素化过程，是26*S*蛋白酶体中泛素化底物高效率降解的重要组成部分^[40]^。同时，它们通过影响靶蛋白稳定性和细胞内信号传导，参与NSCLC的恶性进展^[37]^。Lei等^[41]^发现，在184例NSCLC患者中Rpn11高表达与淋巴结转移、肿瘤原发灶-淋巴结-转移（tumor-node-metastasis, TNM）分期及患者不良预后呈正相关，提示其可作为独立预后因子。Zhang等^[24]^通过癌症基因组图谱（The Cancer Genome Atlas, TCGA）数据库分析，也证实了这一结果。肿瘤抑制因子p53的降解高度依赖于Rpn11^[42,43]^。进一步研究^[24]^发现，敲除Rpn11后，p53的蛋白稳定性显著增加，导致肺腺癌细胞周期阻滞、细胞凋亡和衰老。类似地，敲除Rpn8后，也能增加p53蛋白的表达，进而抑制NSCLC的恶性进展，但其具体作用机制尚不明确^[25]^。值得注意的是，两者协同作用可能构成p53蛋白稳态的双重调控网络。

### 2.3 Rpn1和Rpn2对NSCLC预后及免疫微环境的影响

肿瘤微环境（tumor microenvironment, TME）是肿瘤细胞赖以生存和发展的细胞环境，包括免疫细胞、成纤维细胞、细胞外基质（extracellular matrix, ECM）等^[44]^。免疫微环境的重新编辑能够通过释放促生长信号、中间代谢物和重塑组织结构等途径，在NSCLC的发生、进展和转移中起关键作用^[45]^。由于26*S*蛋白酶体亚基可以通过调控免疫细胞以及ECM进而影响TME的功能紊乱，这可能是目前针对肺癌为主的恶性肿瘤相关免疫治疗效果不佳的重要原因^[46]^。因此，深入研究26*S*蛋白酶体在NSCLC TME中的作用，对提高免疫治疗的效果具有重要的意义。

研究^[35]^表明，Rpn1在肺腺癌中的表达显著升高，与肿瘤转移及不良预后有关。进一步研究^[26]^发现，Rpn1过表达通过抑制p21蛋白，促进NSCLC细胞的增殖与迁移。此外，Rpn1通过与免疫细胞相互作用，促进了包括乳腺癌^[47]^和NSCLC^[48]^在内的多种肿瘤的细胞增殖。Zhang等^[27]^发现，Rpn1表达水平与NSCLC中免疫浸润细胞的类型及浸润程度有关：在肺腺癌中Rpn1促进M0/M1型巨噬细胞极化并抑制B记忆细胞浸润，而在肺鳞癌中Rpn1负向调控CD8^+ ^T细胞及中性粒细胞浸润。这些差异提示，Rpn1可能通过影响免疫细胞浸润，从而影响NSCLC预后。然而，TME是一个复杂的动态系统，Rpn1的具体作用机制仍需进一步研究验证。

ECM是TME的重要组成部分，包含纤维蛋白、多糖和胶原蛋白等。Rpn2通过与ECM-受体相互作用，激活磷脂酰肌醇3激酶/蛋白激酶B（phosphoinositide 3-kinase/protein kinase B, PI3K/Akt）、Wnt、Src-FAK和Rho-GTPases等通路，促进NSCLC的发生和发展^[36]^。此外，Rpn2通过结合配对相关同源盒转录因子2（pair-related homeobox transcription factor 2, PRRX2），调节CD4^+^ T细胞、中性粒细胞和树突状细胞的表达，进而改变TME，提示其可能通过调控ECM和免疫细胞浸润的相互作用，影响NSCLC的预后^[28]^。

### 2.4 Rpt亚基在NSCLC中的研究进展

Rpt4作为19*S*调节颗粒中的一个关键亚基，扮演着“引导”底物进入20*S*核心颗粒进行降解的关键角色。Wnt信号通路对细胞的生长、分化至关重要，轴抑制蛋白1（axis inhibition protein 1, Axin1）是Wnt信号通路的一个重要抑制因子。在NSCLC中Rpt4的表达水平与NSCLC的临床特征（淋巴结转移、肿瘤分期等）密切相关^[49]^。更具体地说，Rpt4通过降解Axin1，进而激活Wnt信号通路，使得NSCLC细胞得以加速生长和转移^[29]^。此外，Rpt家族中的其他成员（如Rpt1^[50]^）在NSCLC中高表达，并与肿瘤分化程度呈正相关，但具体机制有待深入研究。

## 3 20*S*核心颗粒在NSCLC中的研究进展

当泛素标记的底物由19*S*调节颗粒识别并转运至20*S*核心颗粒的肽酶腔后，腔内的PSMB5、PSMB6和PSMB7亚基将底物催化裂解成小的肽段。然而，目前关于20*S*核心颗粒与NSCLC直接相关的研究较少。通过TCGA数据库分析，Li等^[51]^研究发现蛋白酶体*α*7亚基（proteasome subunit *α*7, PSMA7）在肺腺癌中显著高表达，并且患者的总生存期更差。但在异种移植模型中，PSMA7却表现出抑癌作用^[30]^，这可能与PSMA7介导的泛素蛋白酶体系统降解癌基因和抑癌基因在内的降解平衡有关，但其在NSCLC中的作用仍存在争议，需要进一步深入研究。

## 4 26*S*蛋白酶体抑制剂在NSCLC治疗中的应用及临床转化进展

### 4.1 19*S*调节颗粒在NSCLC中的靶向治疗研究进展

近年来研究^[52]^发现19*S*调节颗粒内一些在肿瘤细胞中高表达的亚基，具有除了蛋白酶体本身降解蛋白以外的促癌功能。深入探索其在NSCLC中的调控机制，可为肿瘤治疗提供新靶点。随着对19*S*调节颗粒结构及其分子机制的解析，针对其关键靶点（如泛素受体、去泛素化酶^[53]^）的抑制剂逐渐成为研究热点（表2^[40,54-58]^）。例如，调节颗粒抑制肽-1（regulatory particle inhibitor peptoid-1, RIP-1）通过结合Rpt4，阻碍底物的展开与转运^[54]^，抑制p27降解并诱导G_1_/S期细胞周期阻滞^[55]^；双苄叉基哌啶酮（bisbenzylidinepiperidinone, RA190）与Rpn13结合，导致多泛素化蛋白质的积累，并最终引起细胞死亡。近年来，研究^[56]^发现，利用蛋白水解靶向嵌合体技术，将RA190等多种药物与E3泛素连接酶偶联，对Rpn13进行靶向降解，显著降低耐药性并增强抑瘤效果；8-巯基喹啉（8-thioquinoline, 8TQ）、1,10-菲罗啉（O-phenanthroline, OPA）和硫藤黄素（thiolutin, THL）通过螯合Zn^2+^抑制Rpn11的活性^[40,52]^；小分子探针（如TXS-8）特异性结合Rpn6，有效干扰并破坏26*S*蛋白酶体的组装稳定性^[57]^。尽管上述抑制剂在体内外模型中展现出显著抗肿瘤活性，目前仍缺乏关于19*S*调节颗粒亚基抑制剂的临床试验报道。未来研究需进一步阐明19*S*调节颗粒亚基在NSCLC中的具体作用机制，建立疗效预测模型，并充分评估靶向药物的安全性。

**表 2:** 蛋白酶体抑制剂的作用靶点及机制

Target	Inhibitor	Mechanism	Reference
Rpn11	8TQ, OPA, THL	Inhibition of deubiquitinating enzyme activity	^[40]^
Rpt4	RIP-1	Inhibition of protein unfolding activity of 19*S* regulatory particle	^[54,55]^
Rpn13	RA190	Inhibition of substrate recognition by 19*S* regulatory particle	^[56]^
Rpn6	TXS-8	Integrity of the 26*S* proteasome	^[57]^
PSMB5	Bortezomib, Carfilzomib	Inhibition of protein hydrolyzing activity of 20*S* core particle	^[58]^

8TQ: 8-thioquinoline; OPA: O-phenanthroline; THL: thiolutin; RIP-1: regulatory particle inhibitor peptoid-1; RA190: bisbenzylidinepiperidinone; PSMB5: proteasome subunit beta type 5.

### 4.2 20*S*核心颗粒抑制剂在NSCLC治疗中的进展与挑战

目前，20*S*核心颗粒靶向药物的研发在晶体结构研究的支持下取得了巨大的进步，特别是部分靶向药物已进入NSCLC相关临床试验阶段（表2）。硼替佐米作为靶向PSMB5的可逆性抑制剂，是目前临床研究最深入的蛋白酶体靶向药物，尤其在多发性骨髓瘤的治疗中具有显著疗效。硼替佐米抗肿瘤作用机制具有多维度特征，其主要通过抑制蛋白酶体功能调节细胞内蛋白质的稳态，进而影响细胞周期、细胞凋亡和代谢途径^[58]^。Yang等^[59]^研究发现，硼替佐米通过磷酸化作用降低NSCLC细胞中抗凋亡基因Bcl-2的表达水平，促进肿瘤细胞的凋亡。不幸的是，硼替佐米在晚期肺腺癌患者的临床试验中未观察到客观缓解^[60]^，主要原因是硼替佐米抑制蛋白酶体活性的同时会激活核因子κB（nuclear factor κB, NF-κB）和PI3K/Akt抗凋亡通路，导致肿瘤细胞耐药^[61]^。值得注意的是，Lee等^[62]^研究发现，通过17-丙烯胺-17去甲氧格尔德霉素对NSCLC细胞进行干预后，可同时抑制NF-κB和PI3K/Akt促癌信号通路的激活，从而协同增强硼替佐米的抗肿瘤活性。这提示硼替佐米联合其他药物对治疗NSCLC具有不错的应用前景。进一步的临床研究^[63]^发现，硼替佐米联合多西他赛比单独服用硼替佐米治疗具有更长的中位疾病进展时间（4.0 *vs* 1.4个月）和中位无进展生存期（3.1 *vs* 1.5个月）。然而，部分NSCLC患者在使用硼替佐米治疗后发生了白细胞减少、神经毒性和耐药等毒副作用^[64]^。相比于硼替佐米，第二代蛋白酶体抑制剂卡非佐米的毒副作用更小，且对PSMB5的选择性更高。Yang等^[65]^研究发现，卡非佐米通过蛋白激酶B/转录因子叉头盒蛋白O3a（protein kinase B/forkhead box O3a, AKT/FOXO3a）途径上调生长阻滞和DNA损伤诱导蛋白45（growth arrest and DNA damage-inducible 45, Gadd45）的表达，促使肺腺癌细胞G_2_/M期阻滞和凋亡。另一项研究^[66]^表明，卡非佐米其联合吉西他滨，能有效抑制NSCLC细胞增殖和转移，相关机制仍需进一步研究。此外，Zhou等^[67]^研究发现，卡非佐米联合程序性死亡受体1（programmed cell death protein 1, PD-1）抑制剂可以重塑肿瘤相关巨噬细胞（tumor-associated macrophage, TAM）（M2型巨噬细胞转化为M1型巨噬细胞），从而使更多的CD8^+ ^T细胞浸润并控制体内肿瘤。然而，这些靶向药物由于缺乏合适的生物标志物、联合治疗策略的复杂性以及肿瘤异质性，使其临床应用受到了阻碍。未来的研究应进一步探索这些抑制剂在NSCLC治疗中的潜力，特别是在结合免疫治疗和分子靶向治疗的联合策略以及靶向药物疗效动态监测方面。更重要的是，新型药物的研制应降低耐药和毒副作用的临床应用限制，这将为NSCLC患者提供更加个性化的治疗方案，有望显著改善患者的预后和生存率^[68]^。

## 5 结语与展望

总的来说，26*S*蛋白酶体通过调节关键蛋白的表达或降解，在NSCLC的恶性进展中起到了至关重要的作用。II期临床试验表明，针对蛋白酶体核心颗粒的抑制剂在NSCLC治疗中展现出良好的应用前景，特别是在与常规化疗药物联合使用时表现出显著的抗肿瘤效果。然而，目前仍缺乏充分的临床证据来支持这些发现，且针对26*S*蛋白酶体各亚基在NSCLC相关蛋白相互作用的机制研究也较为有限。因此，未来的研究需要更加全面、深入地探讨26*S*蛋白酶体在NSCLC中的调控机制，并通过系统研究为蛋白酶体抑制剂在NSCLC治疗中的应用提供更为坚实的理论依据，进一步的临床研究将是验证这些抑制剂疗效并推动其临床应用的关键。

## References

[1] Bray F, Laversanne M, Sung H, et al. Global cancer statistics 2022: GLOBOCAN estimates of incidence and mortality worldwide for 36 cancers in 185 countries. CA Cancer J Clin, 2024, 74(3): 229-263. doi: 10.3322/caac.21834 38572751

[2] Han B, Zheng R, Zeng H, et al. Cancer incidence and mortality in China, 2022. J Natl Cancer Cent, 2024, 4(1): 47-53. doi: 10.1016/j.jncc.2024.01.006 39036382 PMC11256708

[3] Song Y, Kelava L, Kiss I. MiRNAs in lung adenocarcinoma: role, diagnosis, prognosis, and therapy. Int J Mol Sci, 2023, 24(17): 13302. doi: 10.3390/ijms241713302 37686110 PMC10487838

[4] Song P, Cui Y. Progress and discussion of perioperative targeted therapy in patients with *EGFR*-mutated resectable non-small cell lung cancer. Zhongguo Feiai Zazhi, 2024, 27(5): 383-390. 38880926 10.3779/j.issn.1009-3419.2024.106.11 PMC11183319

[5] Tang W, Zhou W, Ji M, et al. Role of sting in the treatment of non-small cell lung cancer. Cell Commun Signal, 2024, 22(1): 202-217. doi: 10.1186/s12964-024-01586-x 38566036 PMC10986073

[6] Luo C, Lei M, Zhang Y, et al. Systematic construction and validation of an immune prognostic model for lung adenocarcinoma. J Cell Mol Med, 2020, 24(2): 1233-1244. doi: 10.1111/jcmm.14719 31779055 PMC6991688

[7] Fabijan A, Polis B, Zawadzka-Fabijan A, et al. Domains in action: understanding Ddi1’s diverse functions in the ubiquitin-proteasome system. Int J Mol Sci, 2024, 25(7): 4080-4094. doi: 10.3390/ijms25074080 38612889 PMC11012796

[8] Liu X, Delgado E. A novel role of PSMB9 in endothelial cells and atherosclerosis: beyond its canonical function in immunoproteasome. Acta Pharmacol Sin, 2024, 45(7): 1530-1532. doi: 10.1038/s41401-024-01267-y 38570600 PMC11192834

[9] Collins GA, Goldberg AL. The logic of the 26*S* proteasome. Cell, 2017, 169(5): 792-806. doi: 10.1016/j.cell.2017.04.023 28525752 PMC5609836

[10] Deshaies RJ. Proteotoxic crisis, the ubiquitin-proteasome system, and cancer therapy. BMC Biol, 2014, 12: 94-108. doi: 10.1186/s12915-014-0094-0 25385277 PMC4226866

[11] Gu X, Ma S. Recent advances in the discovery of novel peptide inhibitors targeting 26*S* proteasome. Anticancer Agents Med Chem, 2018, 18(12): 1656-1673. doi: 10.2174/1871520618666180813120012 30101718

[12] Aliabadi F, Sohrabi B, Mostafavi E, et al. Ubiquitin-proteasome system and the role of its inhibitors in cancer therapy. Open Biol, 2021, 11(4): 200390. doi: 10.1098/rsob.200390 33906413 PMC8080017

[13] Bard JAM, Goodall EA, Greene ER, et al. Structure and function of the 26*S* proteasome. Annu Rev Biochem, 2018, 87: 697-724. doi: 10.1146/annurev-biochem-062917-011931 29652515 PMC6422034

[14] Deshmukh FK, Ben-Nissan G, Olshina MA, et al. Allosteric regulation of the 20*S* proteasome by the catalytic core regulators (CCRs) family. Nat Commun, 2023, 14(1): 3126. doi: 10.1038/s41467-023-38404-w 37253751 PMC10229617

[15] Ragwan ER, Kisker FM, Morning AR, et al. Slippery sequences stall the 26*S* proteasome at multiple points along the translocation pathway. Protein Sci, 2024, 33(6): e5034. doi: 10.1002/pro.5034 38801231 PMC11129623

[16] Adolf F, Du J, Goodall EA, et al. Visualizing chaperone-mediated multistep assembly of the human 20*S* proteasome. Nat Struct Mol Biol, 2024, 31(8): 1176-1188. doi: 10.1038/s41594-024-01268-9 38600324 PMC11327110

[17] Mao Y. Structure, dynamics and function of the 26*S* proteasome. Subcell Biochem, 2021, 96: 1-151. doi: 10.1007/978-3-030-58971-4_1 33252727

[18] Liu L, Liu A, Dong J, et al. Proteasome 26*S* subunit, non-ATPase 1 (PSMD1) facilitated the progression of lung adenocarcinoma by the de-ubiquitination and stability of PTEN-induced kinase 1 (PINK1). Exp Cell Res, 2022, 413(2): 113075. doi: 10.1016/j.yexcr.2022.113075 35192838

[19] Ying K, Wang C, Liu S, et al. Diverse Ras-related GTPase DIRAS2, downregulated by PSMD 2 in a proteasome-mediated way, inhibits colorectal cancer proliferation by blocking NF-κB signaling. Int J Biol Sci, 2022, 18(3): 1039-1050. doi: 10.7150/ijbs.68312 35173535 PMC8771839

[20] Xu Y, Wang D, Zhao G. Transcriptional activation of proteasome 26*S* non-ATPase subunit 7 by forkhead box P3 participates in gastric cancer cell proliferation and apoptosis. Bioengineered, 2022, 13(2): 2525-2536. doi: 10.1080/21655979.2021.2018097 35037550 PMC8974172

[21] Spano D, Catara G. Targeting the ubiquitin-proteasome system and recent advances in cancer therapy. Cells, 2023, 13(1): 29-54. doi: 10.3390/cells13010029 38201233 PMC10778545

[22] Zhang J, Ma Q, Yu Q, et al. PSMD3-ILF 3 signaling cascade drives lung cancer cell proliferation and migration. Biol Direct, 2023, 18(1): 18-33. doi: 10.1186/s13062-023-00389-3 37337223 PMC10278297

[23] Lv J, Ma S, Wang X, et al. PSMD12 promotes non-small cell lung cancer progression through activating the Nrf2/TrxR1 pathway. Genes Genomics, 2024, 46(3): 263-277. doi: 10.1007/s13258-023-01484-5 38243044

[24] Zhang L, Xu H, Ma C, et al. Upregulation of deubiquitinase PSMD 14 in lung adenocarcinoma (LUAD) and its prognostic significance. J Cancer, 2020, 11(10): 2962-2971. doi: 10.7150/jca.39539 32226511 PMC7086243

[25] Xu X, Xuan X, Zhang J, et al. PSMD7 downregulation suppresses lung cancer progression by regulating the p53 pathway. J Cancer, 2021, 12(16): 4945-4957. doi: 10.7150/jca.53613 34234864 PMC8247365

[26] Matsuyama Y, Suzuki M, Arima C, et al. Proteasomal non‐catalytic subunit PSMD 2 as a potential therapeutic target in association with various clinicopathologic features in lung adenocarcinomas. Mol Carcinog, 2011, 50(4): 301-309. doi: 10.1002/mc.20632 21465578

[27] Zhang J, Zi R, Hu P, et al. The effect of PSMD 2 expression on the prognosis and immune microenvironment of non-small cell lung cancer. Linchuang Yixue Jinzhan, 2024, 14(1): 846-854.

[28] Liu L, Liu A, Liu X. PRRX2 predicts poor survival prognosis, and promotes malignant phenotype of lung adenocarcinoma via transcriptional activates PSMD1. Transl Oncol, 2023, 27: 101586. doi: 10.1016/j.tranon.2022.101586 36379103 PMC9661514

[29] Zhang JY, Shi KZ, Liao XY, et al. The silence of PSMC 6 inhibits cell growth and metastasis in lung adenocarcinoma. Biomed Res Int, 2021, 2021: 9922185. doi: 10.1155/2021/9922185 34239933 PMC8235974

[30] Zhang HR, Fu HQ, Li YJ, et al. Research progress of proteasome *α*7 subunit in cancers. Guoji Laonian Yixue Zazhi, 2023, 44(3): 343-346.

[31] Wang J, Xiang Y, Fan M, et al. The ubiquitin-proteasome system in tumor metabolism. Cancers (Basel), 2023, 15(8): 2385-2414. doi: 10.3390/cancers15082385 37190313 PMC10136670

[32] Adler J, Oren R, Shaul Y. Depleting the 19*S* proteasome regulatory PSMD1 subunit as a cancer therapy strategy. Cancer Med, 2023, 12(9): 10781-10790. doi: 10.1002/cam4.5775 36934426 PMC10225209

[33] Tomko RJ Jr, Taylor DW, Chen ZA, et al. A single α helix drives extensive remodeling of the proteasome lid and completion of regulatory particle assembly. Cell, 2015, 163(2): 432-444. doi: 10.1016/j.cell.2015.09.022 26451487 PMC4601081

[34] Sun S, Liu S, Zhang Z, et al. Phosphatase UBLCP1 controls proteasome assembly. Open Biol, 2017, 7(5): 170042. doi: 10.1098/rsob.170042 28539385 PMC5451543

[35] Zhao H, Lu G. Prognostic implication and immunological role of PSMD2 in lung adenocarcinoma. Front Genet, 2022, 13: 905581. doi: 10.3389/fgene.2022.905581 35754829 PMC9214243

[36] Mei Y, Yang JP, Lang YH, et al. Global expression profiling and pathway analysis of mouse mammary tumor reveals strain and stage specific dysregulated pathways in breast cancer progression. Cell Cycle, 2018, 17(8): 963-973. doi: 10.1080/15384101.2018.1442629 29712537 PMC6103659

[37] Wang J, Wang T, Feng YK, et al. Deubiquitinating enzyme PSMD7 promotes bladder cancer development: Involvement of RAB1A stabilization. Cell Signal, 2024, 114: 110996. doi: 10.1016/j.cellsig.2023.110996 38040402

[38] Fararjeh AS, Chen LC, Ho YS, et al. Proteasome 26*S* subunit, non-ATPase 3 (PSMD3) regulates breast cancer by stabilizing HER2 from degradation. Cancers (Basel), 2019, 11(4): 527. doi: 10.3390/cancers11040527 31013812 PMC6549480

[39] Cheng CC, Chou KF, Wu CW, et al. EGFR-mediated interleukin enhancer-binding factor 3 contributes to formation and survival of cancer stem-like tumorspheres as a therapeutic target against EGFR-positive non-small cell lung cancer. Lung Cancer, 2018, 116: 80-89. doi: 10.1016/j.lungcan.2017.12.017 29413056

[40] Bustamante HA, Albornoz N, Morselli E, et al. Novel insights into the non-canonical roles of PSMD14/POH1/Rpn 11 in proteostasis and in the modulation of cancer progression. Cell Signal, 2023, 101: 110490. doi: 10.1016/j.cellsig.2022.110490 36241058

[41] Lei J, Liu X, Liu W, et al. The prognostic value of USP14 and PSMD 14 expression in non-small cell lung cancer. Ann Transl Med, 2021, 9(12): 1019-1030. doi: 10.21037/atm-21-2748 34277819 PMC8267284

[42] Gülow K, Tümen D, Kunst C. The important role of protein kinases in the p53 sestrin signaling pathway. Cancers (Basel), 2023, 15(22): 5390-5404. doi: 10.3390/cancers15225390 38001650 PMC10670278

[43] Do Patrocinio AB, Rodrigues V, Guidi Magalhães L. P53: stability from the ubiquitin-proteasome system and specific 26*S* proteasome inhibitors. ACS Omega, 2022, 7(5): 3836-3843. doi: 10.1021/acsomega.1c04726 35155881 PMC8829948

[44] Ciernikova S, Sevcikova A, Stevurkova V, et al. Tumor microbiome-an integral part of the tumor microenvironment. Front Oncol, 2022, 12: 1063100. doi: 10.3389/fonc.2022.1063100 36505811 PMC9730887

[45] Li W, Zhang H, You Z, et al. LncRNAs in immune and stromal cells remodel phenotype of cancer cell and tumor microenvironment. J Inflamm Res, 2024, 17: 3173-3185. doi: 10.2147/JIR.S460730 38774447 PMC11108079

[46] Chen B, Zhu H, Yang B, et al. The dichotomous role of immuno-proteasome in cancer: Friend or foe?. Acta Pharm Sin B, 2023, 13(5): 1976-1989. doi: 10.1016/j.apsb.2022.11.005 37250147 PMC10213805

[47] Xuan DTM, Wu CC, Kao TJ, et al. Prognostic and immune infiltration signatures of proteasome 26*S* subunit, non-ATPase (PSMD) family genes in breast cancer patients. Aging (Albany NY), 2021, 13(22): 24882-24913. doi: 10.18632/aging.203722 34839279 PMC8660617

[48] Bai Y, Zhou L, Zhang C, et al. Dual network analysis of transcriptome data for discovery of new therapeutic targets in non-small cell lung cancer. Oncogene, 2023, 42(49): 3605-3618. doi: 10.1038/s41388-023-02866-5 37864031 PMC10691970

[49] Jia H, Tang WJ, Sun L, et al. Pan-cancer analysis identifies proteasome 26*S* subunit, ATPase (PSMC) family genes, and related signatures associated with prognosis, immune profile, and therapeutic response in lung adenocarcinoma. Front Genet, 2023, 13: 1017866. doi: 10.3389/fgene.2022.1017866 36699466 PMC9868736

[50] Wu WM, Zhao TC, Guo X, et al. Expression of proteasome 26*S* subunit ATPase 2 in non-small cell lung cancer patients and its relationship with prognosis. Zhongliu Yufang Yu Zhiliao, 2020, 33(9): 767-774.

[51] Li Y, Huang J, Sun J, et al. The transcription levels and prognostic values of seven proteasome alpha subunits in human cancers. Oncotarget, 2017, 8(3): 4501-4519. doi: 10.18632/oncotarget.13885 27966459 PMC5354849

[52] Muli CS, Tian W, Trader DJ. Small‐molecule inhibitors of the proteasome’s regulatory particle. Chembiochem, 2019, 20(14): 1739-1753. doi: 10.1002/cbic.201900017 30740849 PMC6765334

[53] Śledź P, Baumeister W. Structure-driven developments of 26*S* proteasome inhibitors. Annu Rev Pharmacol Toxicol, 2016, 56: 191-209. doi: 10.1146/annurev-pharmtox-010814-124727 26738474

[54] Lim HS, Archer CT, Kodadek T. Identification of a peptoid inhibitor of the proteasome 19*S* regulatory particle. J Am Chem Soc, 2007, 129(25): 7750-7751. doi: 10.1021/ja072027p 17536803 PMC2543931

[55] Escobar M, Velez M, Belalcazar A, et al. The role of proteasome inhibition in nonsmall cell lung cancer. J Biomed Biotechnol, 2011, 2011: 806506. doi: 10.1155/2011/806506 21629760 PMC3100637

[56] Song Y, Park PMC, Wu L, et al. Development and preclinical validation of a novel covalent ubiquitin receptor Rpn 13 degrader in multiple myeloma. Leukemia, 2019, 33(11): 2685-2694. doi: 10.1038/s41375-019-0467-z 30962579 PMC6783320

[57] Tian W, Trader DJ. Discovery of a small molecule probe of Rpn-6, an essential subunit of the 26*S* proteasome. ACS Chem Biol, 2020, 15(2): 554-561. doi: 10.1021/acschembio.9b01019 31877015

[58] Sogbein O, Paul P, Umar M, et al. Bortezomib in cancer therapy: mechanisms, side effects, and future proteasome inhibitors. Life Sci, 2024, 358: 123125. doi: 10.1016/j.lfs.2024.123125 39413903

[59] Yang Y, Ikezoe T, Saito T, et al. Proteasome inhibitor PS-341 induces growth arrest and apoptosis of non-small cell lung cancer cells via the JNK/c-Jun/AP-1 signaling. Cancer Sci, 2004, 95(2): 176-180. doi: 10.1111/j.1349-7006.2004.tb03200.x 14965369 PMC11160053

[60] Besse B, Planchard D, Veillard AS, et al. Phase 2 study of frontline bortezomib in patients with advanced non-small cell lung cancer. Lung Cancer, 2012, 76(1): 78-83. doi: 10.1016/j.lungcan.2011.09.006 22186627

[61] Alwahsh M, Farhat J, Talhouni S, et al. Bortezomib advanced mechanisms of action in multiple myeloma, solid and liquid tumors along with its novel therapeutic applications. EXCLI J, 2023, 22: 146-168. doi: 10.17179/excli2022-5653 36998701 PMC10043448

[62] Lee KH, Jang AH, Yoo CG. 17-allylamino-17-demethoxygeldanamycin and the enhancement of PS-341-induced lung cancer cell death by blocking the NF-κB and PI3K/Akt pathways. Am J Respir Cell Mol Biol, 2015, 53(3): 412-421. doi: 10.1165/rcmb.2014-0186OC 25633180

[63] Chua ADW, Thaarun T, Yang H, et al. Proteasome inhibitors in the treatment of non-small cell lung cancer: a systematic review of clinical evidence. Health Sci Rep, 2023, 6(11): e1443-e1457. doi: 10.1002/hsr2.1443 38028684 PMC10643516

[64] Zhang N, Lan R, Chen Y, et al. Identification of KDM4C as a gene conferring drug resistance in multiple myeloma. Open Life Sci, 2024, 19(1): 20220848. doi: 10.1515/biol-2022-0848 38623585 PMC11017188

[65] Yang F, Liu WW, Chen H, et al. Carfilzomib inhibits the growth of lung adenocarcinoma via upregulation of Gadd45a expression. J Zhejiang Univ Sci B, 2020, 21(1): 64-76. doi: 10.1631/jzus.B1900551 31898443 PMC6964995

[66] Drilon A, Schoenfeld AJ, Arbour KC, et al. Exceptional responders with invasive mucinous adenocarcinomas: a phase 2 trial of bortezomib in patients with *KRAS* G12D-mutant lung cancers. Cold Spring Harb Mol Case Stud, 2019, 5(2): a003665. doi: 10.1101/mcs.a003665 30936194 PMC6549573

[67] Zhou Q, Liang J, Yang T, et al. Carfilzomib modulates tumor microenvironment to potentiate immune checkpoint therapy for cancer. EMBO Mol Med, 2022, 14(1): e14502. doi: 10.15252/emmm.202114502 34898004 PMC8749493

[68] Rohondia SO, Ahmed ZSO, Dou QP. Updated review and perspective on 20*S* proteasome inhibitors in the treatment of lung cancer. Curr Cancer Drug Targets, 2020, 20(6): 392-409. doi: 10.2174/1568009620666200226094000 32101123

